# Crystal structure and computational studies of (3*Z*)-4-benzoyl-3-[(2,4-di­nitro­phen­yl)hydrazinyl­idene]-5-phenyl­furan-2(3*H*)-one

**DOI:** 10.1107/S2056989016018600

**Published:** 2016-11-29

**Authors:** Yavuz Köysal, Hakan Bülbül, İlhan Özer İlhan, Nazenin Akın, Necmi Dege

**Affiliations:** a Yesilyurt Demir Celik Vocational School, Ondokuz Mayıs University, TR-55139, Samsun, Turkey; b Department of Physics, Faculty of Arts and Sciences, Ondokuz Mayıs University, TR-55139, Samsun, Turkey; c Department of Chemistry, Faculty of Sciences, Erciyes University, 38039, Kayseri, Turkey

**Keywords:** crystal structure, computational studies, furan derivative, hydrazione, π-π inter­actions

## Abstract

In the mol­ecular structure of the title compound, C_23_H_14_N_4_O_7_, the furan, di­nitro­phenyl and phenyl rings are almost in the same plane (r.m.s. deviation = 0.127 Å), with the benzoyl ring inclined by a dihedral angle of 56.4 (1)° to the three-ring system. A bifurcated intra­molecular N—H⋯(O,O) hydrogen bond is present. In the crystal, adjacent mol­ecules are linked by C—H⋯O hydrogen bonds into chains parallel to [001]. A π–π stacking inter­action between the benzoyl and di­nitro­phenyl moieties contributes to the crystal packing. Theoretical calculations using DFT(B3YLP) methods were used to confirm the mol­ecular structure.

## Abstract 2

The mol­ecular structure of the title mol­ecule was confirmed by theoretical calculations using DFT(B3YLP) methods.

## Chemical context   

Furan-2-3-diones are known heterocyclic starting compounds and show a high reactivity. Due to their characteristics, numerous reports have highlighted their usage in chemistry (Ziegler *et al.*, 1967 ▸; Saalfrank *et al.*, 1991 ▸; Sarıpınar *et al.*, 2000 ▸). In furan-2,3-diones, atoms C2, C3, C5 and C6 represent electrophilic sites of different reactivity and can be used for the construction of condensed heterocyclic systems upon reaction with various nucleophiles and binucleo­philes (Kollenz *et al.*, 1976 ▸; Akçamur *et al.*, 1986 ▸; Akçamur & Kollenz, 1987 ▸). The reactions of substituted furan-2,3-diones with dienophiles in different solvents and at various temperatures have also been studied (Kollenz *et al.*, 1984*a* ▸,*b* ▸). Moreover, derivatives of heterocyclic 2,3-diones which are also *α,β*-unsaturated carbonyl compounds have been found to serve as versatile synthetic equivalents in thermolysis reactions (Fulloon *et al.*, 1995 ▸; El-Nabi & Kollenz, 1997 ▸; Kollenz *et al.*, 2001 ▸), cyclo­addition reactions (Kollenz *et al.*, 1987 ▸) and nucleophilic addition reactions (Kollenz *et al.*, 1977 ▸; Altural *et al.*, 1989 ▸). Several attempts to change functional groups in furan- or pyrrol-2,3-diones and related systems have been reported (Fabian & Kollenz, 1994 ▸; Wong & Wentrup, 1994 ▸).

As part of our studies in this area, we have synthesized the title furan-2,3-dione derivative and report here its mol­ecular and crystal structure.

## Structural commentary   

**Figure 1:**
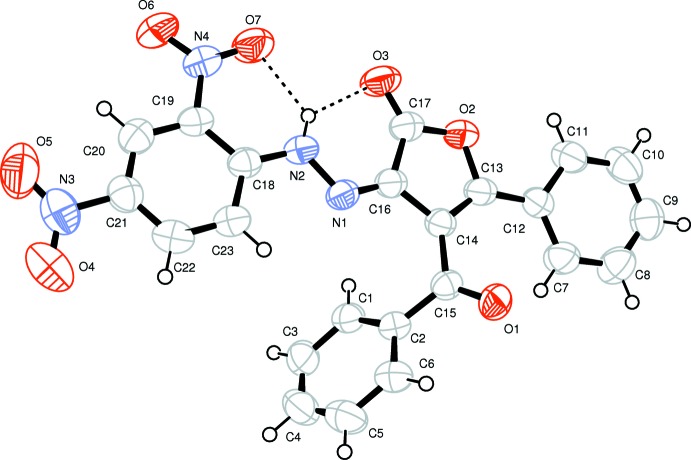
The mol­ecular structure of the title compound, showing the atom-numbering scheme. Displacement ellipsoids are drawn at the 50% probability level. The hydrogen bonds are indicated by dashed lines.

The mol­ecular structure of the title compound is not planar (Fig. 1 ▸). However, three of the four rings, *viz*. C7–C12 (phenyl ring), C13–O2 (furan ring) and C18–C23 (phenyl ring of the di­nitro­phenyl moiety) are almost co-planar. The central furan ring is twisted by 11.30 (5)° to the phenyl ring and by 8.89 (5)° to the di­nitro­phenyl ring. The benzoyl ring is inclined by 56.4 (1)° to the least-squares plane of the three-ring system (r.m.s. deviation = 0.127 Å). Bond lengths and angles for the (2,4-di­nitro­phen­yl)hydrazione moiety are consistent with those in related structures (Fun *et al.*, 2014 ▸; Mague *et al.*, 2014 ▸). The two nitro groups of the di­nitro­phenyl ring are twisted slightly from the ring plane, with torsion angles C22—C21—N3—O4 = −8.1 (3)°, C20—C21—N3—O5 = −9.0 (3)°, C20—C19—N4—O6= − 3.5 (2)° and C18—C19—N4—O7=-4.6 (2)°.
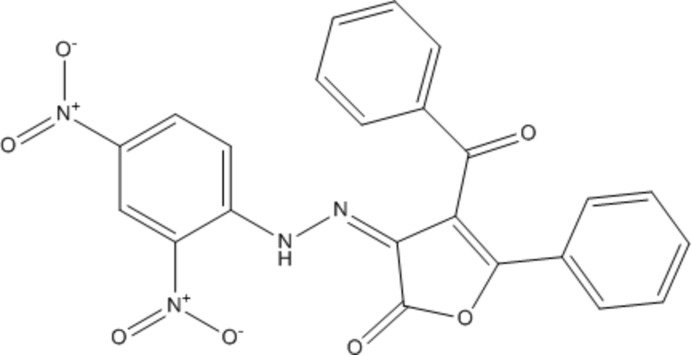


A bifurcated intra­molecular N—H⋯(O,O) hydrogen bond involving both the carbonyl O atom of the furane dione moiety and an O atom of one of nitro groups is present, forming two *S*(6) motifs (Fig. 1 ▸, Table 1 ▸).

## Supra­molecular features   

**Figure 2:**
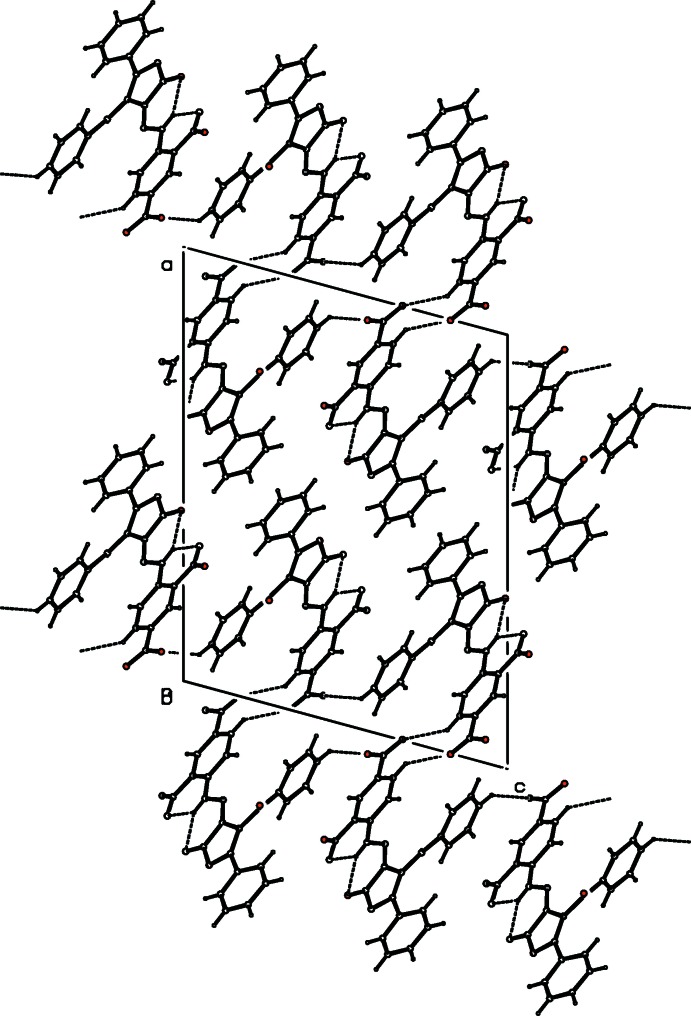
The packing of mol­ecules in the title compound in a view along [010]. Dashed lines indicate C—H⋯O hydrogen bonds.

In the crystal, adjacent mol­ecules are linked through C—H⋯O hydrogen bonds whereby one inter­action (C22—H22⋯O4) leads to a 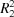
(10) motif and the other (C4—H5⋯O5) links the mol­ecules into chains propagating parallel to [001]. In addition, π–π inter­actions between the C1–C6 [benzoyl; *Cg*(2)] and C18–C23 [di­nitro­phenyl; *Cg*(4)] rings with a centroid-to-centroid distance of *Cg*(2)⋯*Cg*(4)^i^ = 3.81 (1) Å [symmetry code (i) *x*, 3/2-*y*, 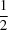 + *z*] are present (Table 1 ▸, Fig. 2 ▸).

## Theoretical calculations   

The mol­ecular structure was optimized using DFT(B3YLP) methods with the 6-31G+(d) basis set (Becke, 1993 ▸; Lee *et al.*, 1988 ▸; Schlegel, 1982 ▸; Peng *et al.*, 1996 ▸) in the calculation and visualization programs of Gaussian03–GaussView4.1 (Frisch *et al.*, 2004 ▸; Dennington *et al.*, 2007 ▸).

The optimized parameters such as bond lengths, bond angles and torsion angles are in good agreement with experimental values on basis of the diffraction study. The highest deviations between the two methods relate to the C4—C5 bond length [1.368 (3) Å from diffraction data, 1.4008 Å from DFT calculations] and the N4—C19—C20—C21 torsion angle [178.85 (14)° from diffraction data, 179.92° from DFT calculations].

**Figure 3:**
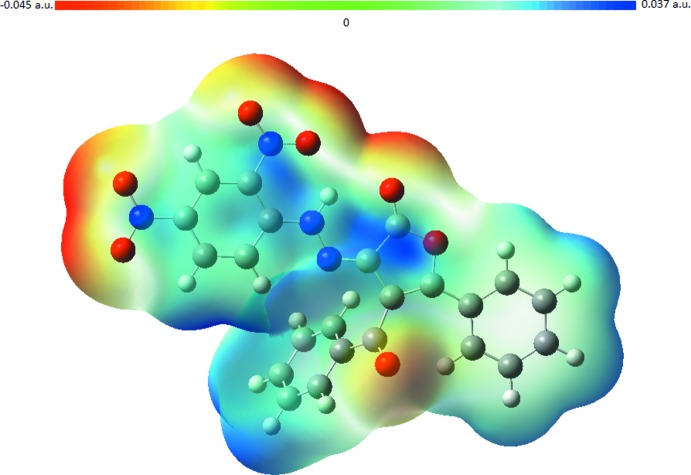
The mol­ecular electrostatic potential surface of the title compound, calculated at the B3LYP/6–31 G+(d) level.

The mol­ecular electrostatic potential is a suitable way to inter­pret the hydrogen-bonding donor and acceptor sides. Electrophilic and nuclecophilic regions are good descriptors for such inter­actions in a mol­ecular electrostatic potential surface. Generally, colours are used for this description. Red-coloured regions are related to a negative electrostatic potential and associated with electrophilic characteristics while blue-coloured regions are related to positive electrostatic potentials and associated with nuclecophilic characteristics. In the title mol­ecule, negative regions are mainly located on atoms O4 and O5 with a minimum value of −0.045 a.u. Positive regions are located around atom N1 with a maximum value of 0.037 a.u. These regions are associated with hydrogen-bonding donor and acceptor sites. The mol­ecular electrostatic potential surface is shown in Fig. 3 ▸.

## Synthesis and crystallization   

A mixture of 4-benzoyl-5-phenyl-2,3-furan­dione (0,5 g., 5,5 mmol) and 2,4-di­nitro­phenyl hydrazine (0,356 g., 5,5 mmol) was dissolved in benzene and stirred about 1 h with a magnetic stirrer. Then the solvent was evaporated and the remaining oily residue was treated with dry diethyl ether and kept at room temperature for 24 h. The precipitate obtained was filtered off and recrystallized from toluene. The completion of the reaction was monitored by TLC. Yield 0,49 g (57%); m.p. = 465 K.

IR (ATR) cm^−1^: 3192.49 (–NH), 3115.20 (aromatic –CH), 1769.25 and 1654.16 (C=O of carbon­yl),1593.73 (C=N of pyrazoline ring), 1493.96 (NO_2_), 1446.99–1334.23 (aromatic C=C) Analysis calculated for C_23_H_14_N_4_O_7_: C,61.57; H,3.87; N, 12.54; found: C, 60.26; H, 3.06; N, 12.23.

## Refinement details   

Crystal data, data collection and structure refinement details are summarized in Table 2 ▸. The H atom attached to the hydrazine group was located from a difference Fourier map and was refined freely. All other H atoms were positioned geometrically and allowed to ride on their parent atoms with C—H = 0.93 Å and *U*_iso_(H) = 1.2*U*_eq_(C).

## Supplementary Material

Crystal structure: contains datablock(s) I. DOI: 10.1107/S2056989016018600/wm5340sup1.cif

Structure factors: contains datablock(s) I. DOI: 10.1107/S2056989016018600/wm5340Isup2.hkl

Supporting information file. DOI: 10.1107/S2056989016018600/wm5340Isup3.cml

CCDC reference: 1514260

Additional supporting information: crystallographic information; 3D view; checkCIF report

## Figures and Tables

**Table 1:** Hydrogen-bond geometry (Å, °)

*D*—H⋯*A*	*D*—H	H⋯*A*	*D*⋯*A*	*D*—H⋯*A*
C4—H4⋯O5^i^	0.93	2.55	3.352 (3)	144
C22—H22⋯O4^ii^	0.93	2.43	3.218 (2)	143
N2—H25⋯O7	0.87 (2)	1.997 (19)	2.6106 (19)	126.8 (16)
N2—H25⋯O3	0.87 (2)	2.118 (19)	2.795 (2)	134.5 (17)

Symmetry codes: (i) 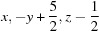; (ii) 
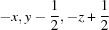
.

**Table 2:** Experimental details

Crystal data	
Chemical formula	C_23_H_14_N_4_O_7_
*M*_r_	458.38
Crystal system, space group	Monoclinic, *P*2_1_/*c*
Temperature (K)	293
*a*, *b*, *c* (Å)	20.7156 (11), 6.3660 (3), 16.0288 (7)
β (°)	105.183 (4)
*V* (Å^3^)	2040.02 (17)
*Z*	4
Radiation type	Mo *K*α
μ (mm^−1^)	0.11
Crystal size (mm)	0.64 × 0.34 × 0.15

Data collection	
Diffractometer	Stoe IPDS 2
Absorption correction	Integration (*X-RED32*; Stoe & Cie, 2002 ▸)
*T*_min_, *T*_max_	0.954, 0.985
No. of measured, independent and observed [*I* > 2σ(*I*)] reflections	18074, 3992, 2617
*R*_int_	0.036
(sin θ/λ)_max_ (Å^−1^)	0.617

Refinement	
*R*[*F* ^2^ > 2σ(*F* ^2^)], *wR*(*F* ^2^), *S*	0.039, 0.089, 1.01
No. of reflections	3992
No. of parameters	311
H-atom treatment	H atoms treated by a mixture of independent and constrained refinement
Δρ_max_, Δρ_min_ (e Å^−3^)	0.11, −0.15

Computer programs: *X-AREA* and *X-RED32* (Stoe & Cie, 2002 ▸), *SHELXS97* (Sheldrick, 2008 ▸), *SHELXL2014* (Sheldrick, 2015 ▸), *ORTEP-3 for Windows* and *WinGX* (Farrugia, 2012 ▸).

## supplementary crystallographic information

### Crystal data

**Table:**

C_23_H_14_N_4_O_7_	*F*(000) = 944
*M**_r_* = 458.38	*D*_x_ = 1.492 Mg m^−^^3^
Monoclinic, *P*2_1_/*c*	Mo *K*α radiation, λ = 0.71073 Å
*a* = 20.7156 (11) Å	Cell parameters from 18113 reflections
*b* = 6.3660 (3) Å	θ = 1.3–27.4°
*c* = 16.0288 (7) Å	µ = 0.11 mm^−^^1^
β = 105.183 (4)°	*T* = 293 K
*V* = 2040.02 (17) Å^3^	Stick, red
*Z* = 4	0.64 × 0.34 × 0.15 mm

### Data collection

**Table:**

Stoe IPDS 2 diffractometer	3992 independent reflections
Radiation source: fine-focus sealed tube	2617 reflections with *I* > 2σ(*I*)
Detector resolution: 6.67 pixels mm^-1^	*R*_int_ = 0.036
ω–scans	θ_max_ = 26.0°, θ_min_ = 2.0°
Absorption correction: integration (*X-RED32*; Stoe & Cie, 2002)	*h* = −25→25
*T*_min_ = 0.954, *T*_max_ = 0.985	*k* = −7→7
18074 measured reflections	*l* = −19→19

### Refinement

**Table:**

Refinement on *F*^2^	0 restraints
Least-squares matrix: full	Hydrogen site location: mixed
*R*[*F*^2^ > 2σ(*F*^2^)] = 0.039	H atoms treated by a mixture of independent and constrained refinement
*wR*(*F*^2^) = 0.089	*w* = 1/[σ^2^(*F*_o_^2^) + (0.0424*P*)^2^] where *P* = (*F*_o_^2^ + 2*F*_c_^2^)/3
*S* = 1.01	(Δ/σ)_max_ < 0.001
3992 reflections	Δρ_max_ = 0.11 e Å^−^^3^
311 parameters	Δρ_min_ = −0.15 e Å^−^^3^

### Special details

**Table:**

Geometry. All esds (except the esd in the dihedral angle between two l.s. planes) are estimated using the full covariance matrix. The cell esds are taken into account individually in the estimation of esds in distances, angles and torsion angles; correlations between esds in cell parameters are only used when they are defined by crystal symmetry. An approximate (isotropic) treatment of cell esds is used for estimating esds involving l.s. planes.

### Fractional atomic coordinates and isotropic or equivalent isotropic displacement parameters (Å^2^)

**Table:**

	*x*	*y*	*z*	*U*_iso_*/*U*_eq_	
N2	0.25724 (8)	0.8002 (2)	0.43332 (9)	0.0568 (4)	
C1	0.21265 (9)	0.5758 (3)	0.18120 (10)	0.0585 (4)	
H1	0.254927	0.632079	0.204818	0.070*	
C2	0.19729 (8)	0.3763 (2)	0.20527 (9)	0.0495 (4)	
C3	0.16531 (11)	0.6908 (3)	0.12232 (11)	0.0738 (6)	
H3	0.175939	0.823402	0.105522	0.089*	
C4	0.10245 (12)	0.6093 (4)	0.08846 (12)	0.0826 (6)	
H4	0.070570	0.687627	0.049082	0.099*	
C5	0.08647 (10)	0.4138 (4)	0.11232 (12)	0.0785 (6)	
H5	0.043669	0.360337	0.089719	0.094*	
C6	0.13366 (9)	0.2962 (3)	0.16968 (10)	0.0616 (4)	
H6	0.122890	0.162162	0.184765	0.074*	
C7	0.37944 (10)	−0.0410 (3)	0.26118 (12)	0.0714 (5)	
H7	0.338362	−0.008307	0.223342	0.086*	
C8	0.41433 (11)	−0.2150 (3)	0.24598 (14)	0.0798 (6)	
H8	0.396471	−0.299188	0.198079	0.096*	
C9	0.47493 (11)	−0.2648 (3)	0.30065 (14)	0.0789 (6)	
H9	0.497450	−0.385144	0.291138	0.095*	
C10	0.50228 (11)	−0.1372 (4)	0.36932 (13)	0.0816 (6)	
H10	0.544155	−0.168646	0.405455	0.098*	
C11	0.46834 (9)	0.0374 (3)	0.38542 (11)	0.0684 (5)	
H11	0.487764	0.123857	0.431993	0.082*	
C12	0.40534 (8)	0.0861 (3)	0.33289 (10)	0.0547 (4)	
C13	0.36878 (9)	0.2643 (3)	0.35482 (9)	0.0541 (4)	
C14	0.30514 (8)	0.3394 (2)	0.32797 (9)	0.0496 (4)	
C15	0.24688 (8)	0.2423 (2)	0.26518 (9)	0.0487 (4)	
C16	0.30221 (9)	0.5239 (2)	0.37966 (9)	0.0519 (4)	
C17	0.36975 (9)	0.5505 (3)	0.43806 (11)	0.0591 (4)	
C18	0.20589 (9)	0.9389 (3)	0.42466 (9)	0.0526 (4)	
C19	0.21248 (8)	1.1297 (3)	0.47072 (9)	0.0541 (4)	
C20	0.16058 (10)	1.2723 (3)	0.45749 (11)	0.0611 (5)	
H20	0.165872	1.397959	0.488098	0.073*	
C21	0.10175 (10)	1.2270 (3)	0.39934 (11)	0.0620 (5)	
C22	0.09279 (9)	1.0401 (3)	0.35364 (11)	0.0663 (5)	
H22	0.052003	1.010889	0.314458	0.080*	
C23	0.14385 (9)	0.8990 (3)	0.36622 (10)	0.0601 (4)	
H23	0.137415	0.773540	0.335401	0.072*	
N1	0.24959 (7)	0.6356 (2)	0.37783 (8)	0.0534 (3)	
N3	0.04722 (10)	1.3806 (3)	0.38399 (13)	0.0822 (5)	
N4	0.27400 (8)	1.1906 (3)	0.53243 (9)	0.0657 (4)	
O1	0.23927 (6)	0.05224 (18)	0.26464 (7)	0.0636 (3)	
O2	0.40819 (6)	0.38975 (19)	0.42049 (7)	0.0630 (3)	
O3	0.39113 (6)	0.6791 (2)	0.49284 (8)	0.0774 (4)	
O4	−0.00184 (9)	1.3482 (3)	0.32419 (12)	0.1127 (6)	
O5	0.05355 (10)	1.5319 (3)	0.43157 (14)	0.1261 (7)	
O6	0.27748 (8)	1.3652 (2)	0.56528 (9)	0.0900 (4)	
O7	0.32091 (7)	1.0674 (3)	0.54951 (9)	0.0941 (5)	
H25	0.2963 (10)	0.827 (3)	0.4674 (12)	0.074 (6)*	

### Atomic displacement parameters (Å^2^)

**Table:**

	*U*^11^	*U*^22^	*U*^33^	*U*^12^	*U*^13^	*U*^23^
N2	0.0586 (10)	0.0590 (9)	0.0480 (7)	−0.0054 (8)	0.0053 (7)	−0.0087 (7)
C1	0.0701 (11)	0.0548 (10)	0.0470 (8)	−0.0028 (9)	0.0087 (8)	−0.0032 (8)
C2	0.0511 (10)	0.0527 (9)	0.0431 (8)	−0.0033 (8)	0.0098 (7)	−0.0047 (7)
C3	0.1018 (17)	0.0596 (11)	0.0551 (10)	0.0135 (11)	0.0117 (11)	0.0042 (9)
C4	0.0831 (16)	0.0984 (17)	0.0576 (11)	0.0325 (13)	0.0029 (10)	0.0014 (11)
C5	0.0558 (12)	0.1085 (17)	0.0647 (11)	0.0069 (12)	0.0041 (9)	−0.0059 (12)
C6	0.0547 (11)	0.0723 (12)	0.0559 (9)	−0.0058 (9)	0.0110 (8)	−0.0060 (8)
C7	0.0543 (11)	0.0804 (13)	0.0734 (12)	0.0042 (10)	0.0059 (9)	−0.0139 (10)
C8	0.0705 (14)	0.0788 (14)	0.0918 (14)	−0.0030 (11)	0.0245 (12)	−0.0223 (11)
C9	0.0775 (15)	0.0734 (13)	0.0930 (14)	0.0137 (11)	0.0348 (12)	0.0041 (12)
C10	0.0689 (13)	0.0996 (16)	0.0738 (12)	0.0259 (12)	0.0145 (10)	0.0118 (12)
C11	0.0615 (12)	0.0827 (13)	0.0570 (10)	0.0075 (10)	0.0082 (9)	0.0017 (9)
C12	0.0530 (10)	0.0575 (10)	0.0514 (9)	0.0002 (8)	0.0096 (8)	0.0045 (8)
C13	0.0563 (11)	0.0573 (10)	0.0439 (8)	−0.0070 (8)	0.0049 (7)	0.0013 (7)
C14	0.0528 (10)	0.0480 (9)	0.0449 (8)	−0.0044 (8)	0.0073 (7)	0.0022 (7)
C15	0.0512 (10)	0.0480 (10)	0.0475 (8)	−0.0062 (8)	0.0139 (7)	−0.0019 (7)
C16	0.0559 (10)	0.0512 (9)	0.0459 (8)	−0.0059 (8)	0.0083 (7)	0.0000 (7)
C17	0.0594 (11)	0.0623 (11)	0.0521 (9)	−0.0070 (9)	0.0081 (8)	−0.0053 (8)
C18	0.0572 (10)	0.0563 (10)	0.0441 (8)	−0.0042 (8)	0.0128 (7)	−0.0004 (7)
C19	0.0589 (10)	0.0588 (10)	0.0434 (8)	−0.0087 (9)	0.0113 (7)	−0.0025 (8)
C20	0.0741 (13)	0.0566 (10)	0.0558 (9)	−0.0038 (10)	0.0228 (9)	−0.0001 (8)
C21	0.0660 (12)	0.0637 (11)	0.0584 (10)	0.0040 (9)	0.0199 (9)	0.0085 (9)
C22	0.0582 (11)	0.0852 (13)	0.0530 (9)	−0.0068 (10)	0.0103 (8)	0.0019 (9)
C23	0.0592 (11)	0.0653 (11)	0.0532 (9)	−0.0068 (9)	0.0101 (8)	−0.0086 (8)
N1	0.0612 (9)	0.0501 (8)	0.0468 (7)	−0.0068 (7)	0.0105 (6)	−0.0050 (6)
N3	0.0786 (13)	0.0811 (13)	0.0892 (12)	0.0119 (11)	0.0261 (10)	0.0214 (11)
N4	0.0695 (11)	0.0699 (11)	0.0560 (8)	−0.0101 (9)	0.0136 (8)	−0.0116 (8)
O1	0.0653 (8)	0.0467 (7)	0.0760 (7)	−0.0090 (6)	0.0136 (6)	0.0004 (6)
O2	0.0558 (7)	0.0680 (8)	0.0564 (6)	−0.0023 (6)	−0.0008 (5)	−0.0081 (6)
O3	0.0701 (8)	0.0847 (9)	0.0679 (7)	−0.0109 (7)	0.0011 (6)	−0.0245 (7)
O4	0.0866 (12)	0.1288 (14)	0.1116 (12)	0.0244 (11)	0.0059 (10)	0.0299 (11)
O5	0.1177 (15)	0.0906 (12)	0.1662 (18)	0.0297 (11)	0.0303 (13)	−0.0205 (12)
O6	0.1058 (12)	0.0720 (9)	0.0814 (9)	−0.0141 (8)	0.0054 (8)	−0.0272 (8)
O7	0.0671 (9)	0.1046 (11)	0.0951 (10)	0.0046 (9)	−0.0063 (8)	−0.0398 (9)

### Geometric parameters (Å, º)

**Table:**

N2—N1	1.3562 (18)	C12—C13	1.457 (2)
N2—C18	1.362 (2)	C13—C14	1.362 (2)
N2—H25	0.87 (2)	C13—O2	1.4007 (18)
C1—C3	1.380 (2)	C14—C16	1.448 (2)
C1—C2	1.388 (2)	C14—C15	1.489 (2)
C1—H1	0.9300	C15—O1	1.2196 (17)
C2—C6	1.389 (2)	C16—N1	1.296 (2)
C2—C15	1.479 (2)	C16—C17	1.475 (2)
C3—C4	1.374 (3)	C17—O3	1.1972 (19)
C3—H3	0.9300	C17—O2	1.370 (2)
C4—C5	1.368 (3)	C18—C23	1.401 (2)
C4—H4	0.9300	C18—C19	1.409 (2)
C5—C6	1.375 (3)	C19—C20	1.380 (2)
C5—H5	0.9300	C19—N4	1.447 (2)
C6—H6	0.9300	C20—C21	1.357 (2)
C7—C8	1.379 (3)	C20—H20	0.9300
C7—C12	1.393 (2)	C21—C22	1.384 (2)
C7—H7	0.9300	C21—N3	1.465 (2)
C8—C9	1.367 (3)	C22—C23	1.362 (2)
C8—H8	0.9300	C22—H22	0.9300
C9—C10	1.366 (3)	C23—H23	0.9300
C9—H9	0.9300	N3—O5	1.214 (2)
C10—C11	1.375 (3)	N3—O4	1.218 (2)
C10—H10	0.9300	N4—O7	1.2230 (19)
C11—C12	1.390 (2)	N4—O6	1.2239 (18)
C11—H11	0.9300		
			
N1—N2—C18	118.69 (14)	C14—C13—C12	135.91 (15)
N1—N2—H25	119.5 (13)	O2—C13—C12	112.81 (14)
C18—N2—H25	121.0 (13)	C13—C14—C16	106.62 (14)
C3—C1—C2	120.12 (17)	C13—C14—C15	127.89 (14)
C3—C1—H1	119.9	C16—C14—C15	125.17 (15)
C2—C1—H1	119.9	O1—C15—C2	120.07 (14)
C1—C2—C6	118.92 (15)	O1—C15—C14	119.81 (14)
C1—C2—C15	122.50 (14)	C2—C15—C14	120.09 (13)
C6—C2—C15	118.55 (15)	N1—C16—C14	126.40 (14)
C4—C3—C1	120.01 (19)	N1—C16—C17	127.16 (15)
C4—C3—H3	120.0	C14—C16—C17	106.34 (15)
C1—C3—H3	120.0	O3—C17—O2	122.55 (16)
C5—C4—C3	120.44 (19)	O3—C17—C16	130.62 (17)
C5—C4—H4	119.8	O2—C17—C16	106.82 (14)
C3—C4—H4	119.8	N2—C18—C23	120.38 (15)
C4—C5—C6	120.03 (19)	N2—C18—C19	122.67 (15)
C4—C5—H5	120.0	C23—C18—C19	116.92 (16)
C6—C5—H5	120.0	C20—C19—C18	121.43 (15)
C5—C6—C2	120.47 (18)	C20—C19—N4	116.10 (15)
C5—C6—H6	119.8	C18—C19—N4	122.44 (16)
C2—C6—H6	119.8	C21—C20—C19	119.29 (16)
C8—C7—C12	120.40 (18)	C21—C20—H20	120.4
C8—C7—H7	119.8	C19—C20—H20	120.4
C12—C7—H7	119.8	C20—C21—C22	121.18 (17)
C9—C8—C7	120.61 (19)	C20—C21—N3	119.20 (18)
C9—C8—H8	119.7	C22—C21—N3	119.61 (18)
C7—C8—H8	119.7	C23—C22—C21	119.83 (17)
C10—C9—C8	119.74 (19)	C23—C22—H22	120.1
C10—C9—H9	120.1	C21—C22—H22	120.1
C8—C9—H9	120.1	C22—C23—C18	121.34 (16)
C9—C10—C11	120.49 (19)	C22—C23—H23	119.3
C9—C10—H10	119.8	C18—C23—H23	119.3
C11—C10—H10	119.8	C16—N1—N2	117.12 (14)
C10—C11—C12	120.77 (18)	O5—N3—O4	124.0 (2)
C10—C11—H11	119.6	O5—N3—C21	118.2 (2)
C12—C11—H11	119.6	O4—N3—C21	117.8 (2)
C11—C12—C7	117.88 (17)	O7—N4—O6	122.19 (16)
C11—C12—C13	119.49 (15)	O7—N4—C19	119.14 (15)
C7—C12—C13	122.62 (15)	O6—N4—C19	118.67 (17)
C14—C13—O2	111.27 (14)	C17—O2—C13	108.93 (13)
			
C3—C1—C2—C6	−0.5 (2)	C15—C14—C16—C17	174.56 (14)
C3—C1—C2—C15	177.30 (15)	N1—C16—C17—O3	−2.5 (3)
C2—C1—C3—C4	1.1 (3)	C14—C16—C17—O3	−179.04 (18)
C1—C3—C4—C5	−0.4 (3)	N1—C16—C17—O2	176.23 (15)
C3—C4—C5—C6	−0.8 (3)	C14—C16—C17—O2	−0.31 (17)
C4—C5—C6—C2	1.4 (3)	N1—N2—C18—C23	7.3 (2)
C1—C2—C6—C5	−0.7 (2)	N1—N2—C18—C19	−170.66 (14)
C15—C2—C6—C5	−178.63 (15)	N2—C18—C19—C20	176.72 (15)
C12—C7—C8—C9	−0.3 (3)	C23—C18—C19—C20	−1.3 (2)
C7—C8—C9—C10	−2.3 (3)	N2—C18—C19—N4	−1.5 (2)
C8—C9—C10—C11	2.1 (3)	C23—C18—C19—N4	−179.50 (14)
C9—C10—C11—C12	0.8 (3)	C18—C19—C20—C21	0.5 (2)
C10—C11—C12—C7	−3.3 (3)	N4—C19—C20—C21	178.85 (14)
C10—C11—C12—C13	176.04 (17)	C19—C20—C21—C22	0.5 (3)
C8—C7—C12—C11	3.1 (3)	C19—C20—C21—N3	−178.66 (14)
C8—C7—C12—C13	−176.23 (17)	C20—C21—C22—C23	−0.7 (3)
C11—C12—C13—C14	−168.59 (18)	N3—C21—C22—C23	178.48 (15)
C7—C12—C13—C14	10.7 (3)	C21—C22—C23—C18	−0.2 (3)
C11—C12—C13—O2	10.6 (2)	N2—C18—C23—C22	−176.95 (15)
C7—C12—C13—O2	−170.12 (15)	C19—C18—C23—C22	1.1 (2)
O2—C13—C14—C16	−0.67 (17)	C14—C16—N1—N2	178.37 (14)
C12—C13—C14—C16	178.51 (17)	C17—C16—N1—N2	2.5 (2)
O2—C13—C14—C15	−174.42 (14)	C18—N2—N1—C16	170.45 (14)
C12—C13—C14—C15	4.8 (3)	C20—C21—N3—O5	−9.0 (3)
C1—C2—C15—O1	−157.59 (15)	C22—C21—N3—O5	171.81 (19)
C6—C2—C15—O1	20.2 (2)	C20—C21—N3—O4	171.09 (17)
C1—C2—C15—C14	24.2 (2)	C22—C21—N3—O4	−8.1 (3)
C6—C2—C15—C14	−158.02 (14)	C20—C19—N4—O7	177.08 (16)
C13—C14—C15—O1	38.1 (2)	C18—C19—N4—O7	−4.6 (2)
C16—C14—C15—O1	−134.56 (16)	C20—C19—N4—O6	−3.5 (2)
C13—C14—C15—C2	−143.63 (16)	C18—C19—N4—O6	174.77 (15)
C16—C14—C15—C2	43.7 (2)	O3—C17—O2—C13	178.77 (16)
C13—C14—C16—N1	−175.98 (15)	C16—C17—O2—C13	−0.08 (17)
C15—C14—C16—N1	−2.0 (2)	C14—C13—O2—C17	0.49 (18)
C13—C14—C16—C17	0.59 (17)	C12—C13—O2—C17	−178.90 (13)

### Hydrogen-bond geometry (Å, º)

**Table:**

*D*—H···*A*	*D*—H	H···*A*	*D*···*A*	*D*—H···*A*
C4—H4···O5^i^	0.93	2.55	3.352 (3)	144
C22—H22···O4^ii^	0.93	2.43	3.218 (2)	143
N2—H25···O7	0.87 (2)	1.997 (19)	2.6106 (19)	126.8 (16)
N2—H25···O3	0.87 (2)	2.118 (19)	2.795 (2)	134.5 (17)

Symmetry codes: (i) *x*, −*y*+5/2, *z*−1/2; (ii) −*x*, *y*−1/2, −*z*+1/2.
